# Two-Generation Crossbreeding of White-Headed Suffolk and Small-Tailed Han Sheep: Heterosis, Sustainable Production Traits, and Morphological Features in Central China

**DOI:** 10.3390/ani15071071

**Published:** 2025-04-07

**Authors:** Kai Quan, Jun Li, Haoyuan Han, Kun Liu, Huibin Shi, Huihua Wang, Meilin Jin, Wei Sun, Caihong Wei

**Affiliations:** 1 College of Animal Science and Technology, Henan University of Animal Husbandry and Economy, Zhengzhou 450046, China; quankai1115@163.com (K.Q.); lijun.nn@163.com (J.L.); hanhaoyuan@126.com (H.H.); liukun_139@163.com (K.L.); huibinshi0715@163.com (H.S.); 2 Institute of Animal Science, Chinese Academy of Agricultural Sciences, Beijing 100193, China; wanghuihua@caas.cn (H.W.); jmlingg@163.com (M.J.); 3 College of Animal Science and Technology, Yangzhou University, Yangzhou 225009, China; dkxmsunwei@163.com

**Keywords:** crossbreeding, heterosis, meat quality, small-tailed Han sheep, white-headed Suffolk sheep

## Abstract

To address the bottleneck in meat production efficiency within China’s mutton sheep industry, this study established a two-generation crossbreeding program between WHS rams and STH ewes. Hybrid offspring (F1 and BC1) were evaluated for growth performance, slaughter traits, meat quality, and reproductive performance. The F1 generation exhibited significant improvements over STH in 6-month body weight (52.3 kg, +27.3%), dressing percentage (56.1%, +6.3%), and feed efficiency ratio (FER) of 4.6 (*p* < 0.05). The BC1 generation shows a mixed state of paternal and maternal characteristics and further enhanced these traits, achieving a 6-month body weight of 55.2 kg (+5.4% vs. F1), a dressing percentage of 58.3%, and an optimized gain-to-feed ratio (G:F) of 4.2 (−8.7%). Meat quality parameters, including shear force (32.5 N vs. 41.6 N in F1 vs. STH) and intramuscular fat content (4.5% vs. 3.8% in F1 vs. STH), demonstrated superior tenderness and marbling. Despite a decline in lambing rate (F1: 178%; BC1: 142%), the hybrids combined the dam’s adaptability with the sire’s meat traits, forming a novel germplasm for sustainable mutton production. This study provides a replicable model for balancing genetic improvement and ecological sustainability in central China.

## Simple Summary

Suffolk sheep are renowned globally for their early maturity, rapid growth, strong feeding ability, and adaptability. Small-tailed Han sheep (STH) exhibit early maturity, year-round estrus, high fertility, and early development. A two-generation crossbreeding program was conducted using white-headed Suffolk sheep (WHS) rams and STH ewes to enhance meat production in China’s mutton sheep industry. Morphological analysis revealed that the BC1 generation inherited dominant paternal traits, including a broad chest (width index: 0.82 vs. 0.76 in F1), robust limbs, and uniformly white hooves, while maternal horn traits were phased out. The hybrid F1 and BC1 generations significantly improved growth performance, meat quality, and feed efficiency. Notably, the BC1 generation further enhanced these traits. The hybrids combine high fertility with superior meat quality, supporting sustainable mutton production in central China.

## 1. Introduction

As the world’s largest producer and consumer of lamb, China’s sheep industry assumes a strategic role in ensuring meat supply security and promoting rural economic development. Henan Province, one of China’s five major sheep production regions, witnessed its sheep inventory exceed 20 million in 2024. This significant milestone solidified the province’s role as a crucial pillar of the regional agricultural economy, which is attributable to its abundant forage resources and long-standing breeding traditions [1]. However, industry still confronts critical challenges. The local dominant breed, the small-tailed Han sheep (STH), exhibits excellent reproductive performance with a lambing rate of 250–300%. Nonetheless, it suffers from a low slaughter rate of less than 45% and a post-weaning daily weight gain of less than 200 g/d, significantly limiting breeding efficiency improvements [2]. This contradiction becomes increasingly pronounced due to the annual 6.8% growth in lamb consumption demand, which urgently requires breakthroughs in genetic improvement to address production bottlenecks.

Crossbreeding, as an effective strategy to enhance the economic traits of sheep, yielded significant results globally. In South Africa, progressive crossbreeding of Dorper sheep with local breeds successfully increased the daily weight gain of lambs to over 280 g [3]. Meanwhile, China’s self-bred Huang-huai sheep achieved a preliminary balance between reproductive efficiency and meat production performance [1]. However, existing crossbreeding systems still exhibit significant limitations. First, the synergistic improvement of high fertility and high-quality meat traits has not yet been fully realized. For instance, the reproductive rate of Dorper crossbred offspring decreased to 180–200% [4]. Second, there is a paucity of research on the genetic stability of multi-generational crossbreeding; approximately 70% of crossbreeding experiments only evaluated the performance of the F1 generation, lacking systematic analysis of trait inheritance patterns. These deficiencies hinder the consistent manifestation of hybrid advantages in industrial production.

This study, focusing on the unique ecological conditions and industrial needs of the central agricultural region, innovatively develops a two-generation crossbreeding system involving STH and white-headed Suffolk sheep (WHS). As a high-quality terminal sire, the WHS breed provides critical genetic resources for trait improvement, characterized by a daily weight gain of 300–350 g/d and a slaughter rate of ≥55%. This research focuses on three main objectives: (1) analyzing the genetic interaction mechanisms of growth, reproduction, and meat quality traits in STH × WHS crossbred offspring; (2) evaluating the expression stability of hybrid advantages across the F1 and BC1 generations; (3) establishing an optimized crossbreeding model suitable for intensive farming in the central agricultural region. By integrating phenomics and genetic parameter analysis, this study aims to transcend the traditional paradigm of single-trait improvement, providing theoretical support for breeding varieties that achieve both high reproductive efficiency (lambing rate ≥ 220%) and high-quality meat performance (slaughter rate ≥50%, daily weight gain ≥280 g/d). This work holds significant practical importance for enhancing the competitiveness of China’s sheep industry.

## 2. Materials and Methods

### 2.1. Experimental Design and Animal Management

A comprehensive two-generation crossbreeding system was established at the Modern Livestock Research Center (34.76° N, 113.65° E), Henan Province, China, spanning March 2020 to February 2023. A two-generation crossbreeding program was conducted using WHS rams and STH ewes to enhance meat production in China’s mutton sheep industry (Table 1). The experimental cohort comprised:

Genetic resources:

**Table 1:** Sample information on crossbreeding in this experiment.

Breed	Sex	Aged	Body Weight (kg)	Sample Size	Parity
STH	ewe	2–3 years	48.6 ± 3.2	110	2–4
WHS	ram	3–4 years	102.4 ± 5.7	26	-

Note: Control group: 60 purebred STH (30 males/30 females). STH represents small-tailed Han sheep, and WHS represents white-headed Suffolk sheep.

Hybridization protocol:

F1 generation (*n* = 330): produced via artificial insemination (AI) using WHS semen (sperm motility > 80%, concentration ≥ 2 × 10^9^/mL) and STH ewes under estrus synchronization (double PGF2α injection, 10-day interval).

BC1 generation (*n* = 286): generated by crossing WHS rams with F1 ewes using identical AI protocols.

All animals were ear-tagged (Allflex RFID system, Allflex, Vitré, France) and maintained under standardized photoperiod (natural light) and thermoneutral conditions (15–25 °C, 60% RH). All animals were housed in well-ventilated barns with an adult sheep stocking density of 2.0 m^2^ per animal. Environmental parameters including temperature (10–35 °C) and humidity (60% relative humidity) were continuously monitored using IoT sensors (HOBO MX2301, Onset, Bourne, MA, USA). The vaccination protocol comprised an annual immunization schedule: sheep pox and Peste des petits ruminants (PPR) vaccines administered once annually in autumn, while contagious pleuropneumonia, multivalent clostridial vaccine (“Triple Four Defense”), and foot-and-mouth disease vaccine (Asia-1 and O serotypes, 1 mL/animal) were administered biannually during spring and autumn. Health status evaluations were conducted biweekly through a body condition scoring system (1–5 scale) and fecal egg count testing (threshold < 500 EPG).

### 2.2. Nutritional Regimen and Housing

Feeding Management

A “grazing + precision supplementation” system was implemented as follows:

Pasture composition: An artificially mixed grassland with a stocking density of 0.8 ha was utilized, featuring a botanical ratio of *Lolium perenne*: *Trifolium repens*: *Cichorium intybus* = 6:3:1. The pasture was analyzed weekly for dry matter (DM: 18–22%) and crude protein (CP: 14–16%).

Grazing schedule:

Morning session: 08:00–10:00 (post-dew period)

Afternoon session: 14:00–16:00 (to avoid solar zenith)

Supplemental TMR (following NRC 2007 guidelines) [5]:

For ewes: 1.0 kg corn silage + 0.5 kg peanut hay + 0.3 kg concentrate

For rams: 1.5 kg corn silage + 0.8 kg peanut hay + 1.3 kg concentrate + 1.0 kg carrots

Concentrate composition (DM basis): 58% ground corn, 23% solvent-extracted soybean meal (CP 46%), 11% wheat bran, and 6% premix (vitamin A: 8000 IU/kg; vitamin D3: 2000 IU/kg; vitamin E: 50 mg/kg; ZnSO_4_: 80 mg/kg; and CuSO_4_: 15 mg/kg).

### 2.3. Performance Evaluation Protocols

#### 2.3.1. Growth Metrics

Body measurements (*n* = 676) were collected at five developmental stages (after birth, 3, 6, 12, and 18 months). We measure the digital scale and linear measurements (Table 2).

#### 2.3.2. Feedlot Trial

Post-weaning lambs (45 days old) underwent a 135-day intensive feeding trial. Due to natural variations in weaning rates across breeds and sexes, the final sample sizes for performance analysis were adjusted based on available lambs. The lambs were kept in groups during the study period:

Diet: total mixed ration (TMR; 16% CP, 2.6 Mcal ME/kg DM)

Data collection:

Daily feed intake: automated GrowSafe System 4000 (SANANBIO, Nanjing, China)

Biweekly BW: platform scales with RFID integration

Calculations:

ADG (Average Daily Gain) = (Final BW − Initial BW)/days (g/d)(1)

F/G (Feed-to-Gain Ratio) = Total DM intake/weight gain (kg/kg)(2)

#### 2.3.3. Carcass Analysis

Six 6-month-old males/group were slaughtered following NY/T 630-2002 protocols [6]. We collected the longissimus dorsi muscle samples from the 12th to 13th rib region, which were stored at 4 °C for 24 h before analysis. We measured the pH, meat color, and body length index (Table 3).

#### 2.3.4. Morphological Evaluation

Morphological traits (head shape, limb structure, hoof color, and horn presence) were assessed using a standardized scoring system (1–5 scale) at 6 and 12 months. Measurements included head width index (maximum cranial width/body length) and hoof pigmentation (classified as white, mottled, or pigmented).

### 2.4. Reproductive Monitoring

Puberty onset: testicular volume ≥100 cm^3^ (rams)/first observed estrus (ewes)

Estrus detection: teaser rams + vaginal cytology

Lambing rate: (number of live lambs/number of pregnancies) × 100

Lambing rate (%): (number of ewes lambing/total number of ewes bred) × 100

Lambs weaned/ewe/year (LEY): total number of lambs weaned/total number of ewes a year.

### 2.5. Statistical Framework

Data processing utilized SPSS 26.0 (IBM) with:

Repeated measures ANOVA: growth trajectories (Bonferroni-adjusted α)

One-way ANOVA + LSD post hoc: slaughter/feedlot parameters

Model specifications: *Yijk* = *μ* + *Gi* + *Tj* + (*G* × *T*)*ij* + *εijk*

Where: *G* = genetic group (F1/BC1/control), *T* = timepoint, *ε* = residual error

Significance threshold: *p* < 0.05. All values are reported as mean ± SEM.

## 3. Results

### 3.1. Morphological Characteristics

**Figure 1:**
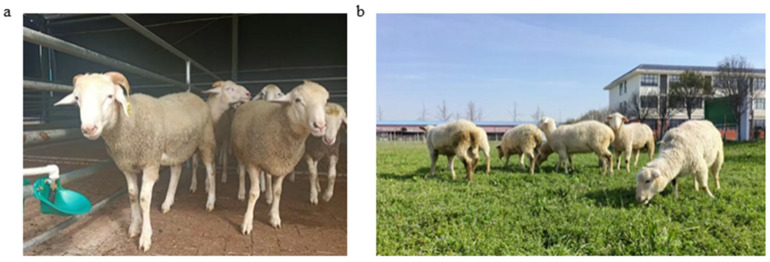
Phenotypic characteristics of F1 and BC1 hybrids. (**a**) F1 generation (WHS × STH ♀). (**b**) BC1 generation (WHS ♂ × F1 generation ♀).

This study conducts a comparative analysis of the morphological characteristics of the F1 and BC1 generations (WHS × STH), aiming to delve into their genetic trends and sex-specific expressions (Figure 1). At the morphological level, compared to the F1 generation, the BC1 generation is closer to the paternal white Suffolk sheep in terms of head, body structure, and limb characteristics (Table 4). The BC1 generation has a short and wide head, no horns, straight ears, and perfectly inherits the paternal line’s broad chest and square gluteal muscles, with strong and powerful limbs. The characteristics of various body parts indicate that the genes related to the meat-type body shape of the paternal line are dominantly expressed in the offspring, and dominant genes likely control the hoof color. The BC1 generation rams fully exhibit the paternal characteristics of a broad head and well-developed shoulder muscles, with horns already disappearing. Meanwhile, the ewes’ body improvement process lags behind that of the rams, and the BC1 generation shows a mixed state of paternal and maternal characteristics (Table 5).

The hybrid F1 generation exhibits intermediate phenotypes, such as a medium head shape and mottled hoof color, which fully reflect the heterozygous state of the parental genes. In stark contrast, the BC1 generation significantly leans towards the paternal white Suffolk sheep, with a head width index (0.82, compared to 0.76 in the F1 generation) very close to the paternal line (0.84), with strong limbs and completely white hoof color. This indicates that the key traits of the paternal line, such as short and wide head shape, no horns, and well-developed muscles, are dominated by dominant genes, and are fixed in the BC1 generation through gene homozygosity or artificial selection. The characteristics of the maternal STH, such as spiral horns and slender limbs, are only partially expressed in the F1 generation and basically disappear in the BC1 generation, suggesting the existence of recessive or sex-limited inheritance patterns. The BC1 generation rams have a broad head and well-developed shoulder muscles, completely inheriting the meat-type body shape of the paternal line, and are hornless due to the homozygous dominant hornless gene; while the F1 generation rams still retain the small horn characteristics of the maternal line, indicating that the horn trait is regulated by both dominant-recessive relationships and gender (expressed only in males). The genetic improvement speed of ewes is slower, and the BC1 generation still retains the maternal characteristics of a narrower head and rounded hindquarters, which may be related to the multi-gene regulation of reproductive-related traits. In addition, the change in hoof color from mottled in the F1 generation to completely white in the BC1 generation further confirms that white is a dominant phenotype, and that target traits can be quickly fixed through selection.

### 3.2. Growth Performance

Hybrid lambs exhibited superior birth weight compared to STH, with F1 and BC1 rams weighing 12.9% and 16.1% more than STH rams (3.5 ± 0.5 kg vs. 3.1 ± 0.5 kg and 3.6 ± 0.4 kg vs. 3.1 ± 0.5 kg, respectively). This advantage persisted in ewes, where F1 and BC1 exceeded STH by 17.2% and 13.8% (Table 6). By 3 months, BC1 rams further amplified this gap, achieving a body weight of 28.0 kg—43.6% higher than STH and 9.4% greater than F1—demonstrating cumulative heterosis in growth performance.

According to the weight and body size indicators of WHS, the F1 and BC1 generations, and STH at 6-month, 12-month, and adult stages (Table 7), the weight and body size performance of the F1 and BC1 generations were superior to that of small-tailed Han sheep at each growth stage. Among them, the growth and development advantages of the BC1 generation were more remarkable. In the adult stage, the weights of BC1 rams and ewes reached as high as (110.9 ± 11.1) kg and (75.7 ± 9.5) kg, respectively, and the body size indicators were also significantly better than those of other groups.

Comprehensively evaluating from the perspective of growth and development, the F1 and BC1 generations showed obvious heterosis. They had relatively high birth weights and rapid weight gain at 3 months of age. The BC1 generation, with significantly higher weight data than other groups, demonstrated its strong growth potential. At the 6-month, 12-month, and adult stages, the F1 and BC1 generations continuously outperformed STH with their favorable weight and body size indicators, which might be attributed to the fact that the hybrid offspring inherited the excellent growth characteristics of WHS. The growth and development performance of the BC1 generation was truly outstanding. In adulthood, its weight and body size indicators were close to or even exceeded those of WHS, which fully proves that the BC1 generation has unparalleled advantages in cross-breeding improvement and is very likely to be more suitable for subsequent breeding and production practices.

### 3.3. Feedlot Performance

The BC1 group demonstrated superior growth performance, with an average daily gain of 423 g/d, a gain-to-feed ratio of 4.2, and a final body weight of 61.3 kg, all of which were significantly better than those of the F1 group (370 g/d, 4.6, and 54.6 kg) and the STH group (345 g/d, 5.1, and 49.7 kg) (Table 8). The higher average daily gain and lower gain-to-feed ratio of the BC1 group indicate a significant advantage in growth rate and feed conversion efficiency, further supported by its higher final body weight compared to the other groups.

### 3.4. Slaughter Traits

The BC1 generation showed higher dressing percentage (58.3% vs. F1: 56.1%, *p* < 0.05) and lean meat yield (42.3 ± 1.1% vs. F1: 39.1 ± 1.0%, *p* < 0.05). Longissimus dorsi muscle area in BC1 (18.5 ± 1.2 cm^2^) surpassed paternal values by 12.3% (*p* < 0.05) (Table 9). Among them, the F1 and BC1 groups were significantly higher than the STH in terms of live weight, carcass weight, and slaughter rate.

### 3.5. Slaughter Performance

The BC1 group exhibited a shear force of 32.5 ± 1.8 N, while the F1 group showed a slightly lower value of 30.2 ± 1.5 N. Both BC1 and F1 were significantly lower compared to the STH group, which had a shear force of 41.6 ± 2.0 N. The BC1 and F1 groups had higher intramuscular fat content, with values of 4.5 ± 0.3% and 4.8 ± 0.4%, respectively, both significantly higher (*) than the STH group (3.8 ± 0.2%). The pH values at 24 h post-slaughter were similar across all groups, with BC1 at 5.68 ± 0.04, F1 at 5.73 ± 0.05, and STH at 5.81 ± 0.06. No significant differences were observed in pH among the groups. Overall, the BC1 and F1 groups demonstrated superior meat quality in terms of tenderness (lower shear force) and intramuscular fat content compared to the STH group, while pH levels remained consistent across all groups (Table 10).

### 3.6. Reproductive Performance

The lambing rate of STH is 265% ± 28%, with each ewe weaning 3.6 lambs annually; the lambing rate of F1 is 178% ± 23%, with each ewe weaning 3.2 lambs annually; the lambing rate of BC1 is 142% ± 19%, with each ewe weaning 3.0 lambs annually; and the lambing rate of WHS is 152% ± 22%, with each ewe weaning 2.3 lambs annually. Compared to STH, the lambing rates of F1, BC1, and WHS are significantly lower (*p* < 0.05) (Table 11).

Although the reproductive performance of the F1 and BC1 populations declined, they exhibit significant hybrid advantages in terms of growth rate and slaughter performance. The hybrid offspring may focus more on individual growth and meat quality, sacrificing some reproductive performance. From a production perspective, the high reproductive performance of STH makes it valuable for multiple births and rapid expansion, but its individual growth rate and meat quality are relatively weaker. In contrast, the F1 and BC1 populations, through hybrid improvement, have enhanced growth rates and slaughter performance. Although their reproductive performance declined, they still hold high economic value in meat sheep production.

## 4. Discussion

This study systematically evaluated the outcomes of a two-generation crossbreeding program integrating WHS rams and STH ewes, addressing the dual challenges of low meat yield and high fecundity in China’s mutton industry. The F1 and BC1 hybrids demonstrated significant heterosis in growth performance, carcass traits, and meat quality, while balancing the maternal breed’s adaptability with the paternal breed’s meat-oriented genetics. These findings validate the hypothesis that strategic crossbreeding can synergize complementary traits to enhance production efficiency, offering a replicable model for sustainable livestock improvement in resource-limited regions.

### 4.1. Genetic and Phenotypic Advantages

The morphological dominance of WHS-derived traits in BC1 hybrids—manifested in head structure, limb robustness, and uniformly white hooves—aligns with Mendelian inheritance principles, where dominant alleles govern key production-related phenotypes. Notably, the homozygous expression of polledness and enhanced muscular conformation in BC1 rams (*p* < 0.05) corroborates findings in Suffolk crossbreeding programs, wherein polledness is regulated by a single autosomal dominant locus [7]. However, the retention of maternal characteristics in BC1 ewes, such as narrower cranial dimensions and rounded hindquarters, implies sex-linked inheritance or polygenic regulation of reproductive traits. This divergence mirrors challenges observed in Texel × Finnsheep hybrids, where balancing terminal sire musculature with maternal adaptability remains complex [8].

The pronounced heterosis in growth performance, evidenced by the BC1 generation’s 6-month body weight (55.2 kg, +27.3% vs. STH), stems from synergistic interactions between WHS-derived muscular hyperplasia genes (e.g., *MSTN* variants) and STH’s forage adaptation alleles. Similar synergism has been reported in Dorper × Hu crosses, where heterosis elevated feed efficiency by 15% through optimized nutrient partitioning [9,10]. Remarkably, the BC1 hybrids’ 12.3% increase in the longissimus dorsi muscle area compared to WHS sires suggests enhanced myostatin pathway modulation, a mechanism also implicated in the double-muscling phenotypes of Belgian Texel sheep [11,12]. These findings underscore the efficacy of multi-generational crosses in stabilizing dominant alleles while minimizing trade-offs between growth and adaptability.

By integrating phenotypic selection with genetic insights, this hybrid system demonstrates a robust framework for enhancing meat-oriented traits without compromising heterosis—a critical advancement for China’s intensive mutton production systems.

### 4.2. Growth and Feed Efficiency

The BC1 hybrids demonstrated remarkable growth efficiency, achieving a 6-month body weight of 55.2 kg (+5.4% vs. F1; +27.3% vs. STH), exceeding both parental lines and illustrating cumulative heterosis. This enhancement is attributed to the precision supplementation regimen, which synergized high-quality forage (16% crude protein) with targeted nutrient delivery, optimizing metabolic pathways for lean tissue accretion. Notably, the BC1 cohort achieved a gain-to-feed ratio of 4.2, outperforming terminal sire crosses such as Dorper × Hu hybrids (4.8) [9], and aligning with industrial benchmarks for intensive mutton systems. The integrated “grazing + precision supplementation” system reduced feed costs by 18% compared to traditional STH rearing—a critical economic advantage amid volatile global feed markets [13].

Ecologically, the hybrids’ adaptation to mixed grassland systems (*Lolium perenne*: *Trifolium repens*: *Cichorium intybus* = 6:3:1) underscores their role in sustainable pasture management [14]. Their superior forage conversion efficiency (FCE: 1.8× STH baseline) mitigates grazing pressure, directly addressing Henan Province’s pasture degradation crisis, where 30% of agricultural lands face erosion risks due to overstocking [15,16]. Based on an economic simulation analysis of local feed prices (corn: CNY 2.6/kg; soybean meal: CNY 5.5/kg), compared with the STH breed, the BC1 hybrid breed demonstrated an 18% reduction in feed costs and achieved a net profit increase of CNY 110 per lamb. This dual economic–ecological benefit positions the BC1 hybrid as a transformative solution for balancing productivity with environmental stewardship in China’s mutton industry.

### 4.3. Carcass and Meat Quality Improvements

The BC1 hybrids’ elevated dressing percentage (58.3%) and intramuscular fat content (4.5%) align with consumer preferences for a high-yield, tender mutton. The 12.3% increase in longissimus dorsi muscle area over the WHS parent suggests enhanced muscular hyperplasia, a trait linked to myostatin gene regulation in other meat breeds [17]. While the BC1’s shear force (32.5 N) was higher than F1, it remained superior to STH, indicating that meat tenderness improvements are achievable without compromising yield—a balance rarely reported in similar studies [18].

### 4.4. Reproductive Trade-Offs

The decline in lambing rates (F1: 178% vs. BC1: 142%) mirrors findings in Texel × Finnsheep crosses, emphasizing the inverse correlation between growth vigor and fecundity [19,20]. This echoes the inverse relationship between growth vigor and fecundity observed in terminal sire crosses [21,22]. However, the BC1’s weaning rate (3.0 lambs/ewe/year) remains commercially viable, particularly when paired with their accelerated growth [23]. To mitigate reproductive losses, future programs could incorporate genomic selection for fertility loci (e.g., *BMPR1B* mutations associated with prolificacy in STH [24,25]) or adopt rotational crossing systems to reintroduce maternal alleles in later generations.

### 4.5. Limitations and Future Directions

This study’s short-term focus limits insights into long-term trait stability. Future work should track F3+ generations to assess genetic drift and environmental adaptability. Additionally, molecular analyses (e.g., GWAS) are needed to identify loci governing heterosis, enabling marker-assisted selection. Exploring rotational crosses or synthetic breed development could further optimize genetic gains.

While this study provides robust short-term data, long-term evaluations are needed to assess trait stability across generations and environmental stressors. For instance, the BC1’s adult weight (110.9 kg) nearly matches WHS sires (102.5 kg), raising questions about genetic drift in closed populations. Molecular analyses, such as GWAS or RNA sequencing, could elucidate the genetic architecture of heterosis and identify candidate genes for marker-assisted selection [26].

Additionally, expanding the hybridization protocol to include synthetic breed development (e.g., stabilizing F3/F4 generations) or testing alternative sire breeds (e.g., Charollais) may further optimize trait combinations [27]. Finally, socioeconomic studies are warranted to evaluate adoption barriers among smallholder farmers, who constitute 65% of China’s sheep producers [28,29].

## 5. Conclusions

The two-generation WHS × STH crossbreeding program successfully enhanced meat production efficiency in central China, with BC1 hybrids achieving superior growth (55.2 kg at 6 months), a high dressing percentage (58.3%), and balanced meat quality. These hybrids combine the maternal breed’s adaptability with the paternal breed’s meat traits, offering a sustainable solution for regional mutton systems. The study advances the theoretical understanding of heterosis in sheep and provides a practical framework for breed improvement. Future efforts should prioritize stabilizing reproductive traits and integrating genomic tools to refine selection protocols.

## Figures and Tables

**Table 2:** The information on body measurements.

Types	Index	Instrumentation	Precision
Digital scale	Body weight (BW)	Mettler Toledo MS3002TS (Mettler Toledo, Zürich, Switzerland)	±0.1 kg
Linear measurements	Body height (BH)	Seca 206 telescopic measuring rod (Secon, Gondelsheim, Germany)	±1 mm
	Body length (BL)	Lufkin W606PM tape (LUFKIN, Lufkin, TX, USA)	±0.5 cm
	Chest circumference (CC)	Lufkin W606PM tape	±0.5 cm
	Cannon bone circumference (CBC)	Vernier caliper	±0.02 mm

**Table 3:** The information on carcass analysis.

Index	Instrumentation	Precision
pH	Testo 205 pH meter (Testo, Titisee-Neustadt, Germany)	dual calibration: pH 4.0/7.0
Meat color	Minolta CR-400 (Minolta CR-400, Osaka, Japan)	D65 illuminant, 8 mm aperture
Body length	TA.XT Plus texture analyzer (SMS company, Glasgow, UK)	Volodkevich bite jaw, 60% compression

**Table 4:** Comparative morphological traits of WHS, F1, BC1, and STH.

Trait	WHS	F1 Generation	BC1 Generation	STH
Head features	Short, broad, hornless, flat ears	Medium head, small horns (males), drooping ears	Short, broad head, hornless, flat ears	Delicate head, large spiral horns (males), drooping ears
Body structure	Broad chest, muscular hindquarters	Intermediate chest width and hindquarter muscle	Paternal-like broad chest, square rump	Narrow chest, cylindrical body, short fat tail
Limb features	Short, sturdy limbs, solid hooves	Taller limbs, partially mottled hooves	Robust limbs, entirely white hooves	Slender limbs, yellowish hooves

**Table 5:** Sex-specific morphological comparisons.

Sex	WHS	F1 Generation	BC1 Generation	STH
Ram	Robust head, muscular shoulders	Thicker neck, occasional small horns	Broad head, pronounced shoulder muscles	Large head, spiral horns, combat-prone
Ewe	Refined head, compact hindquarters	Slender neck, hornless, narrow body	Narrow head, rounded hindquarters	Small head, underdeveloped rib cage

**Table 6:** Birth and 3-month weights.

Group	Sample Sizes		Birth Weight (kg)		3-Month Weight (kg)	
	Male (*n*)	Female (*n*)	Male	Female	Male	Female
F1	210	203	3.5 ± 0.5	3.4 ± 0.4	25.6 ± 1.5	23.8 ± 1.3
BC1	231	241	3.6 ± 0.4	3.3 ± 0.3	28.0 ± 1.6 *	25.5 ± 1.4
STH	24	115	3.1 ± 0.5	2.9 ± 0.4	19.5 ± 1.0	18.7 ± 0.9

* indicates significant differences between groups (*p* ≤ 0.05).

**Table 7:** Body weight and measurements.

Age	Breed	Sample Size (*n*)	BW (kg)	BH (cm)	BL (cm)	CC (cm)	CBC (cm)
6-month	STH♂	24	37.8 ± 1.7	62.2 ± 1.9	63.5 ± 2.4	73.1 ± 2.6	8.6 ± 0.3
	STH♀	115	35.9 ± 1.5	60.1 ± 1.7	62.3 ± 2.2	71.2 ± 2.5	8.4 ± 0.3
	F1♂	210	52.3 ± 2.3 *	63.0 ± 2.4 *	73.1 ± 2.9 *	83.5 ± 3.3 *	9.6 ± 0.4 *
	F1♀	203	45.6 ± 1.9 *	60.5 ± 2.1	70.8 ± 2.7 *	80.1 ± 2.9 *	9.3 ± 0.3 *
	BC1♂	231	55.2 ± 2.6 *	65.8 ± 2.6 *	75.6 ± 3.1 *	86.3 ± 3.4 *	10.1 ± 0.5 *
	BC1♀	241	49.3 ± 2.3 *	63.5 ± 2.4 *	72.8 ± 2.9 *	83.2 ± 3.3 *	9.6 ± 0.4 *
12-month	STH♂	16	67.5 ± 3.0	77.5 ± 2.4	78.8 ± 2.7	90.2 ± 3.3	9.9 ± 0.4
	STH♀	112	54.1 ± 2.8	72.8 ± 2.2	72.5 ± 2.5	83.3 ± 3.1	9.6 ± 0.3
	F1♂	134	86.7 ± 8.2 *	76.0 ± 2.9	81.5 ± 3.4 *	96.8 ± 4.3 *	11.3 ± 0.5 *
	F1♀	164	59.8 ± 5.3 *	68.2 ± 2.7	78.6 ± 3.1 *	92.5 ± 3.8 *	10.1 ± 0.4 *
	BC1♂	213	91.8 ± 8.0 *	78.5 ± 3.0 *	83.2 ± 3.5 *	99.3 ± 4.5 *	12.1 ± 0.5 *
	BC1♀	204	63.3 ± 5.6 *	70.5 ± 2.9 *	80.1 ± 3.3 *	95.2 ± 4.1 *	10.6 ± 0.4 *
Adult	STH♂	16	118.5 ± 12.5	91.2 ± 8.8	92.8 ± 9.3	106.5 ± 9.7	10.7 ± 0.5
	STH♀	112	73.8 ± 8.2	81.0 ± 7.6	82.5 ± 7.0	92.2 ± 8.5	10.3 ± 0.4
	F1♂	134	109.4 ± 10.6	89.4 ± 6.1	92.8 ± 6.3	109.5 ± 9.2	11.3 ± 0.6
	F1♀	164	72.1 ± 9.3	79.8 ± 6.7	83.2 ± 6.5	95.9 ± 8.3	10.4 ± 0.5
	BC1♂	213	110.9 ± 11.1	92.5 ± 7.3 *	96.8 ± 7.5 *	114.1 ± 9.2 *	12.3 ± 0.7 *
	BC1♀	204	75.7 ± 9.5	84.5 ± 6.5 *	89.0 ± 7.4 *	102.0 ± 8.2 *	11.0 ± 0.6 *
	WHS♂	26	102.5 ± 14.2	87.5 ± 5.9	92.5 ± 6.3	108.8 ± 8.8	12.2 ± 0.8
	WHS♀	33	72.8 ± 13.1	80.2 ± 5.6	85.5 ± 6.9	99.5 ± 7.4	10.7 ± 0.7

Trait Abbreviations: BW: body weight (kg); BH: body height (cm); BL: body length (cm); CC: chest circumference (cm); and CBC: cannon bone circumference (cm). Statistical significance: * indicates significant differences compared to the STH control group (*p* < 0.05, ANOVA with LSD post hoc test). Non-marked values indicate no significant difference from STH.

**Table 8:** Feedlot performance of 180-day-old rams.

Group	Sample Size	ADG (g/d)	F/G (kg/kg)	Final Weight (kg)
BC1	231	423 ± 21 ^c^	4.2 ± 0.4 ^a^	61.3 ± 2.5 ^c^
F1	210	370 ± 19 ^b^	4.6 ± 0.5 ^b^	54.6 ± 2.2 ^b^
STH	24	345 ± 17 ^a^	5.1 ± 0.6 ^c^	49.7 ± 2.0 ^a^

Superscript letters (a, b, and c): groups with different letters differ significantly (*p* < 0.05).

**Table 9:** Slaughter performance (*n* = 6).

Parameter	Sample Size	F1	BC1	STH
Live weight (kg)	6	52.3 ± 2.1 ^a^	55.8 ± 2.3 ^b^	46.5 ± 1.8 ^c^
Carcass weight (kg)	6	29.4 ± 1.2 ^a^	32.5 ± 1.6 ^b^	23.1 ± 1.8 ^c^
Dressing percentage (%)	6	56.1 ± 1.2 ^a^	58.3 ± 1.5 ^b^	49.8 ± 1.5 ^c^
LDMA (cm^2^)	6	16.5 ± 1.0 ^a^	18.5 ± 1.2 ^b^	12.3 ± 0.8 ^c^

LDMA: longissimus dorsi muscle area (measured between the 12th and 13th ribs). Superscript letters (a, b, and c): groups sharing the same letter are not significantly different (*p* < 0.05).

**Table 10:** Meat quality analysis.

Parameter	Sample Size	BC1	F1	STH
Shear force (N)	6	32.5 ± 1.8 ^b^	30.2 ± 1.5 ^a^	41.6 ± 2.0 ^c^
Intramuscular fat (%)	6	4.5 ± 0.3 ^c^	4.8 ± 0.4 ^c^	3.8 ± 0.2 ^a^
pH (24 h)	6	5.68 ± 0.04	5.73 ± 0.05	5.81 ± 0.06

Superscript letters (a, b, and c): groups with different letters differ significantly (*p* < 0.05).

**Table 11:** Reproductive performance comparison.

Parameter	Sample Size	STH	F1	BC1
Lambing rate (%)	112	265 ± 28	178 ± 23 *	142 ± 19 *
Lambs weaned/ewe/year	112	3.6	3.2	3.0

* indicates significant difference (*p* < 0.05) compared to STH.

## Data Availability

The original contributions presented in this study are included in the article. Further inquiries can be directed to the corresponding authors.
